# Unmet needs with antipsychotic treatment in schizophrenia and bipolar I disorder: patient perspectives from qualitative focus groups

**DOI:** 10.1186/s12888-023-04746-4

**Published:** 2023-04-12

**Authors:** Michael J. Doane, Kimberly Raymond, Cory Saucier, Leona Bessonova, Amy K. O’Sullivan, Michelle K. White, April Mitchell Foster, Kaitlin LaGasse, Julia Carpenter-Conlin, Martha Sajatovic, Dawn I. Velligan

**Affiliations:** 1 Alkermes, Inc., Waltham, MA USA; 2 QualityMetric, Inc. LLC, Johnston, RI USA; 3 University Hospitals Cleveland Medical Center, Cleveland, OH USA; 4 The University of Texas Health Science Center at San Antonio, San Antonio, TX USA

**Keywords:** Antipsychotics, Drug side effects, Focus groups, Patient involvement, Patient preference

## Abstract

**Background:**

Schizophrenia (SZ) and bipolar I disorder (BD-I) are chronic mental health disorders often treated with antipsychotic medications. This qualitative study sought to better understand disease burden and treatment experiences with oral antipsychotic medications in participants living with SZ or BD-I.

**Methods:**

Six 90-min focus groups were conducted with participants diagnosed with SZ or BD-I. Trained moderators facilitated discussions using a semistructured guide. Participants described symptoms, impacts of disease, and experiences with oral antipsychotic medications, whether favourable or unfavourable.

**Results:**

Among participants with SZ (*n* = 15; 3 groups, 5 per group), 53% were male and 33% were white, with a mean of 18.6 years since diagnosis. Of participants with BD-I (*n* = 24; 3 groups, 8 per group), 33% were male and 42% were white, with a mean of 13.0 years since diagnosis. Participants described numerous symptoms of their illnesses that impacted relationships and daily life, including effects on emotional health, the ability to work, and encounters with law enforcement. Previous antipsychotic medications were deemed effective by 14/15 (93%) participants with SZ and 12/16 (75%) participants with BD-I. Most participants with SZ (13/15; 87%) or with BD-I (16/24; 67%) reported discontinuing their antipsychotic medication at some point. Side effects were a common reason for discontinuing or switching medications for participants with SZ (8/15; 53%) and for those with BD-I (11/24; 46%). The most common side effects reported in both cohorts were weight gain, drowsiness, sexual problems, and neurologic symptoms. Side effects negatively affected quality of life, leading to serious health problems and issues with self-esteem.

**Conclusions:**

People living with SZ or BD-I cited a range of favourable and unfavourable experiences with oral antipsychotic medications. Most participants reported that their antipsychotics were effective at controlling their symptoms, but multiple side effects impacted their quality of life, caused additional serious health problems, and often led to discontinuation of or switching antipsychotics. Findings from this study contribute to a better understanding of patients’ experiences with antipsychotics and highlight a need for new medications with favourable benefit/risk profiles.

## Introduction

Schizophrenia (SZ) and bipolar I disorder (BD-I) are chronic and debilitating mental health disorders. Both disorders are associated with substantial disease burden, including the presence of medical and psychiatric comorbidities, the potential for lifelong disability, and the risk of premature mortality [1–4]. As a consequence, people living with SZ or BD-I have increased health care resource use and associated medical costs [5, 6]. Additionally, symptoms of SZ and BD-I adversely impact employment and work productivity and negatively affect relationships and quality of life [7, 8].

Antipsychotic medications are effective at reducing the positive symptoms of SZ [9, 10] and are often used with mood stabilisers to treat manic or depressive episodes of BD-I and to provide maintenance therapy to prevent relapse [11, 12]. For both patient populations, long-term antipsychotic treatment is associated with better clinical outcomes, reduced disease burden, improved quality of life, and lower health care costs [10, 13–17].

Despite their effectiveness, antipsychotic medications are associated with side effects, including weight gain and metabolic disturbances, sedation/somnolence, sexual dysfunction, amenorrhea and galactorrhoea, and neurologic symptoms (eg, parkinsonism or akathisia), that may add to the underlying disease burden [11, 18–20]. Many people find these side effects a hinderance to daily functioning [21, 22], contributing to suboptimal medication adherence and/or treatment discontinuation, which are common across both conditions [21–23], increasing the risk of hospitalisation and associated direct medical costs [16, 24–26].

Understanding the impact of antipsychotic medication use, and the treatment needs that remain unmet for people living with SZ or BD-I, is essential for optimising treatment effectiveness, improving medication adherence, and reducing disease burden. Previous research indicates that antipsychotic treatment has negative impacts on social and work life in people with SZ [21] or BD-I [22]. However, because of the lack of patient perspective data on the experience of taking antipsychotic medications, the extent of these impacts is unclear. Focus groups allow participants to provide detailed information that conventional clinical study assessments and other quantitative research may not capture [27]. This qualitative study used focus groups to explore treatment experiences with oral antipsychotic medications among people with a self-reported physician diagnosis of SZ or BD-I.

## Methods

### Research design

This qualitative, cross-sectional study used focus groups to characterise facets of disease burden and treatment experiences of individuals diagnosed with SZ or BD-I. Previous research suggests that the inclusion of 5 to 10 participants per focus group [28] and utilisation of 4 groups [29] is sufficient to achieve saturation, ie, the point at which no new concepts emerge from iterative rounds of interviews. In-person focus groups were conducted in August 2018 at two sites (Fort Lee, NJ, USA, and Atlanta, GA, USA). Disease burden and treatment experiences with oral antipsychotic medications were discussed in three 90-min focus groups among participants living with SZ (*n* = 15; 5 participants per group). Likewise, participants living with BD-I participated in three separate 90-min focus groups (*n* = 24; 8 participants per group). In addition to the qualitative information obtained from focus groups, participants were asked to rate the effectiveness of their preferred antipsychotic medication on a scale of 1 (not effective) to 10 (very effective) and to rate their level of satisfaction with their current antipsychotic medication on a scale of 1 (not satisfied) to 5 (very satisfied).

### Participant sample

This study was approved by the New England Independent Review Board, and written informed consent was obtained from all participants. Participants with varied demographic characteristics, including age, sex, race/ethnicity, and education, were recruited by a third-party vendor from existing survey panels. Individuals interested in participating in the study completed an online screening survey. Eligibility was then determined via a telephone interview. Eligible individuals were at least 18 years of age, with a self-reported diagnosis of SZ or BD-I received from a mental health care professional (MHCP) at least 12 months prior to recruitment. Potential participants were required to have taken an oral medication for SZ or BD-I within the previous 12 months, with ongoing treatment under the care of an MHCP for at least 3 months, and to be fluent in English. Individuals with SZ were excluded if they were experiencing active psychosis; those with BD-I were excluded if they also reported having a diagnosis of SZ. Hospitalisation for psychiatric care within the previous 3 months was exclusionary for both groups.

### Data collection and analysis

Two moderators experienced in qualitative research facilitated discussions in each focus group, and a clinical nurse was present at each location for safety purposes. Moderators used a semi-structured interview guide that was reviewed by expert clinicians, representatives from leading mental health patient advocacy organisations, and an independent review board. The interview guide contained open-ended, exploratory questions to allow for spontaneous responses. Probing questions were used to clarify information and to elicit additional responses on concepts of interest.

Participants were asked about their symptoms, impacts of disease on relationships and daily functioning, perceptions of antipsychotic medications, and decisions related to medication use and adherence. Given the semi-structured nature of focus groups, responses were collaborative and not standardised across groups. After completion of the focus group, each participant received $300 as compensation for their time and travel.

Participant responses were digitally recorded, and audio recordings were transcribed verbatim and checked for accuracy. Transcripts were coded independently by the 2 moderators using content thematic analysis [30] in accordance with grounded theory methodology [31], whereby concepts emerge from participants rather than from an a priori theory. Respective disease experiences provided by each group of participants were reviewed by the moderators; emerging themes and subthemes were identified and tracked. Between-moderator coding discrepancies were reviewed, discussed, and resolved by the research team. Qualitative data were content coded and analysed using NVivo qualitative research software version 11 (QSR International; Burlington, MA, USA). Overarching themes such as the impact of disease on relationships and work life, as well as participants’ treatment experiences, were spontaneously expressed by participants and recorded. For both SZ and BD-I, saturation (ie, no new themes identified) was achieved after the third focus group.

## Results

### Participant characteristics

A total of 15 participants with SZ and 24 participants with BD-I completed the study. As noted in Table 1, the mean (standard deviation [SD]) age of participants with SZ was 49.1 (9.1) years, with a mean (SD) time since diagnosis of 18.6 (11.4) years. Among the focus group participants with SZ, 8 (53%) were male, 5 (33%) were white, and 7 (47%) had obtained an associate degree or higher college education. The mean (SD) age of participants with BD-I was 47.5 (14.5) years, with a mean (SD) time since diagnosis of 13.0 (9.0) years. Of the focus group participants with BD-I, 8 (33%) were male, 10 (42%) were white, and 18 (75%) had an associate degree or higher college education.

**Table 1:** Demographics and baseline characteristics

Parameter	Schizophrenia (*n* = 15)	Bipolar I Disorder (*n* = 24)
Age, mean (SD)	49.1 (9.1)	47.5 (14.5)
Time since diagnosis, mean (SD), years	18.6 (11.4)	13.0 (9.0)
**Sex, n (%)**		
Female	7 (46.7)	16 (66.7)
Male	8 (53.3)	8 (33.3)
**Race, n (%)**		
Black	7 (46.7)	9 (37.5)
Asian	1 (6.7)	3 (12.5)
White	5 (33.3)	10 (41.7)
Other	2 (13.3)	2 (8.3)
**Ethnicity, n (%)**		
Hispanic	4 (26.7)	4 (16.7)
**Education, n (%)**		
Less than high school diploma	3 (20.0)	0 (0)
High school diploma or General Education Diploma	2 (13.3)	5 (20.8)
Some college	3 (20.0)	1 (4.2)
Associate degree or technical certificate	3 (20.0)	6 (25.0)
Bachelor’s degree	3 (20.0)	8 (33.3)
Graduate degree	1 (6.7)	4 (16.7)

### Reported conditions and symptoms related to SZ or BD-I

Comorbid psychiatric conditions endorsed and disease symptoms experienced by participants are displayed in Table 2. For participants with SZ, self-reported psychiatric conditions included depression, posttraumatic stress disorder, and bipolar disorder, whereas participants with BD-I self-reported substance use disorder, posttraumatic stress disorder, and anxiety disorder.

**Table 2:** Self-Reported comorbid psychiatric conditions and disease-related symptoms experienced over the course of illness

Parameter	Participants
**Participants With Schizophrenia (*n* = 15)**	
Self-reported psychiatric conditions, n (%)	
Depression	7 (47)
Posttraumatic stress disorder	3 (20)
Bipolar disorder	3 (20)
Disease-related symptoms, n (%)	
Paranoia/suspicion	13 (87)
Auditory hallucinations	12 (80)
Anxiety	11 (73)
Cognitive difficulties	8 (53)
Depression	8 (53)
Delusions	8 (53)
Visual hallucinations	7 (47)
Anger	6 (40)
Negative thoughts	5 (33)
**Participants With Bipolar I Disorder (*n* = 24)**	
Self-reported psychiatric conditions, n (%)	
Substance use disorder	7 (29)
Posttraumatic stress disorder	6 (25)
Anxiety disorder	5 (21)
Disease-related symptoms, n (%)	
Depression	16 (67)
Mania	13 (54)
Impulsive or excessive spending	11 (46)
Suicidal thoughts	10 (42)
Racing thoughts	8 (33)
Anger	6 (25)
Anxiety	6 (25)
Episodic cycling	6 (25)
Auditory or visual hallucinations	6 (25)
Sexual promiscuity or hypersexuality	6 (25)
Risk taking	5 (21)
Difficulty sleeping	5 (21)

Participants with SZ reported 14 discrete symptoms related to disease burden that were experienced in the course of their illness, the most common being paranoia/suspicion, auditory hallucinations, and anxiety. Other symptoms reported by at least 5 participants with SZ were cognitive difficulties, depression, delusions, visual hallucinations, anger, and negative thoughts. Participants with BD-I reported 37 discrete disease-related symptoms during their illness, with depression, mania, and impulsive or excessive spending as the 3 most common. Other symptoms reported by at least 5 participants with BD-I were suicidal thoughts, racing thoughts, anger, anxiety, episodic cycling, auditory or visual hallucinations, sexual promiscuity or hypersexuality, risk taking behaviour, and difficulty sleeping.

### Unfavourable impacts of SZ or BD-I symptoms on quality of life

Focus group discussions elicited a variety of responses from participants, who described in detail how symptoms of their respective disease affected their quality of life. Illustrative quotes from participants with SZ are presented in Table 3, and those for participants with BD-I are presented in Table 4.

**Table 3:** Quality-of-life impacts among participants with schizophrenia^a^

**Personal and social relationships**	“I said to her, ‘I still love you. Do you still love me?’ And she said, she started crying, and she said, ‘But you’re mentally ill.’ So that was the problem with our relationship, because of my mental illness.”
	“Everything you do that people do normally is a challenge. Building relationships, schizophrenia stole that from me.”
	“I don’t allow myself to get in relationships anymore.”
	“It’s hard for [my family]. They don’t understand the illness. They said, ‘You did this. You put me through this.’ But they don’t understand that it wasn’t me. It was the illness.”
**Emotional health**	“Sometimes I say, ‘Only time I’m gonna be free is when I die.’ And that’s sad, but that’s the only time I’m gonna be free of this illness.”
	“I’m to the point now, you know, I don’t – I don’t share [about my illness] … I’ve had people say to me, ‘Are you crazy?’”
**Ability to work**	“I just couldn’t keep up or I couldn’t focus, and some days I was too scared to even go to work because I thought something was going to happen to me on the way there.”
	“When I was at work I was afraid people were whispering about me and I got paranoid.”
**Encounters with law enforcement**	“My mother used to watch kids at the house. And I put my hand over the kid’s mouth. I was sick at the time, and they called the police and then I was in the hospital.”

^a^Responses edited for clarity

**Table 4:** Quality-of-life impacts among participants with bipolar I disorder^a^

**Personal and social relationships**	“Not only is ending relationships a problem for me, but sometimes, having healthy relationships is a problem because I end up attracting the wrong kind of people.”
	“I try to stay away from relationships.”
	“But as far as personal relationships, [they] just never worked out for me. They haven’t lasted more than three to six months. Once I get depressed, I don’t return calls. I, you know, isolate.”
	“I still have sort of like social phobia.”
**Emotional health**	“Because if I over-commit, get into too many things, my head starts working on two things, I’ll feel anxiety coming up in my body. And I know that my anxiety gets too high, I can’t function. I can’t do anything. I can’t make good decisions.”
	“Because of my symptoms I have that anxiety.”
	“I was drinking more frequently than I ever have during that period, just doing a lot of self-medicating.”
**Ability to work**	“I lost my job over 2 years ago. I’m still looking for another job. It was related to bipolar [disorder]. I just, I couldn’t go to work.”
**Encounters with law enforcement**	“I had suicide thoughts real bad. I was crying, I had called the police on myself.”
	“I starting thinking that people were living under my bed. They were living in my attic. I had called the police a couple times. And told them to go up in the attic to my living [room] up in the attic, and they was saying, we have spider webs up there, nobody’s up there.”

^a^Responses edited for clarity

#### Relationships and emotional health

Participants often stated that symptoms of SZ or BD-I negatively impacted their relationships and emotional health. Twelve participants (80%) with SZ reported that their symptoms got in the way of relationships with family members or significant others, generally due to lack of trust and/or paranoia/suspiciousness. While discussing how SZ affected relationships, one woman stated:

*“I had a lot of problems [with] my marriage, my daughter … and [it] affected my husband a lot emotionally and I lost a lot of time in a sense … my mother, my parents … it affected them. It was just a lot for them to deal with.”*

According to 9 participants (60%), stigma associated with SZ was the most challenging aspect of their condition, while 10 participants (67%) struggled to maintain their sense of self. A man with SZ described the stigma that he experienced during a family holiday as follows:

*“You go on Thanksgiving and [my sister] just yelled out, she says, ‘What happened to him? He didn’t take his medication today?’ And everybody’s laughing. I felt like this small. I was just so embarrassed … I have little nieces and nephews, and they’ll say, ‘Oh, you’re crazy.’ You get labelled crazy. It’s just hard … people can accept if you have cancer better than they can accept you having this illness.”*

Similarly, 21 participants with BD-I (88%) reported that their relationships were substantially impacted by the disease, with 5 (21%) reporting that they intentionally sabotaged relationships to keep people away. Eight participants (33%) cited damage to relationships due to compulsive spending, gambling, and sexual promiscuity associated with manic episodes. When discussing how mania led to a loss of trust, a man with BD-I stated:

“Yeah. A lot to cover up what I’m doing, like gambling or overspending. I go on spending binges, too. So that’s – that feeds my mania.”

Eleven participants with BD-I (46%) recounted the stigma of being referred to as mentally ill and its effect on their relationships and interactions with other people. While discussing how BD-I affected family interactions, a woman stated:

“My family thought I was using it as a crutch … You feel kind of rejected, too, when you tell people things, and you know, they hold it against you or don’t believe you.”

#### Ability to work

Symptoms related to SZ or BD-I had a substantial impact on participants’ abilities to work. Ten participants with SZ (67%) reported that their symptoms had impacted their ability to sustain employment, and 8 (53%) were unemployed, while 6 (40%) were on long-term disability. Three participants (20%) reported having delusions about co-workers, while 2 (13%) noted that trouble concentrating made it difficult or impossible to get work done. A man with SZ stated that stress was a factor in his decision to take a lower-paying job:

*“I had a position that was kind of high stress, so I took a step down in the company for [a] job with lesser stress. Even though I make less money, I’m not as stressed out and snappy and not able to … present myself in a way that I should.”*

Participants with BD-I also reported that symptoms affected their work lives. Ten participants (42%) were unemployed, while 7 (29%) had lost a job because of their symptoms, such as mood swings or impulsive decisions. In addition, 6 (25%) participants quit their jobs because of BD-I symptoms or hospitalisations. When discussing the impact of BD-I symptoms on work, a woman explained:

“Whenever I’ve been hospitalised, I’m way too embarrassed to go back [to work]. So, I just don’t.”

#### Encounters with law enforcement and the judicial system

Encounters with law enforcement were commonly discussed among focus group participants. Six participants (40%) with SZ reported having criminal or legal problems, while 3 (20%) referenced past incarceration. One woman described in detail how her symptoms of SZ led to her incarceration:

*“I go into a parking garage … I just start, like, screaming out … like a siren noise I was making to alarm everybody that the world was about to end. And then … people upstairs … they could hear me and they called the police. I tried to explain to them … But instead of them taking me directly to a hospital, they took me to jail.”*

Twelve participants (50%) with BD-I reported being arrested or incarcerated and attributed those instances to their behaviour and poor decision-making during a manic or depressive episode. According to a woman with BD-I:

*“It was too much with the new family, new baby, new job, new house … I had an episode … I almost crash[ed] my car, but I didn’t. But I got arrested for that … They thought I was having a DUI [driving under the influence], because I was just acting so drunk.”*

### Favourable impacts of oral antipsychotic medications

Most focus group participants with SZ (*n* = 10; 67%) or BD-I (*n* = 22; 92%) reported taking antipsychotic medications. Eight participants (53%) with SZ described treatment with antipsychotics as successful overall. When describing the favourable effects of oral antipsychotic medications, participants with SZ indicated that they were calmer and less agitated (*n* = 6; 40%), less paranoid (*n* = 5; 33%), and more focused (*n* = 3; 20%). However, 3 participants (20%) were still seeking effective medications. Ten participants (42%) with BD-I indicated that their most effective medication alleviated symptoms such as depression, mania, anxiety, mood swings, racing or suicidal thoughts, and sleep issues.

When asked to rate the effectiveness of their oral antipsychotic medication on a scale of 1 (not effective) to 10 (very effective), most participants with SZ (*n* = 14; 93%) rated their medication a 6 or higher. In addition, participants reported high satisfaction with their current medication, with an average rating of 4.4 on a scale of 1 (not satisfied) to 5 (very satisfied). Due to time constraints, participants in 2 BD-I focus groups (*n* = 16) were asked to rate only antipsychotic medication effectiveness, whereas participants in 1 BD-I focus group (*n* = 8) were asked to rate only their satisfaction with their current oral antipsychotics. Most participants with BD-I (*n* = 12; 75%) thought that their medication was effective, rating it a 6 or higher, and overall, participants were satisfied with their current oral antipsychotic medication, with 5 (63%) reporting a score of 4 or higher.

According to one woman with BD-I, her oral antipsychotic medication alleviated several symptoms of her disease:

“The med combo I’m on right now … has definitely helped with the mania and the depression, suicidal thoughts. And I’m not self-harming anymore.”

In addition, a woman with SZ described how the effectiveness of controlling symptoms with her current medication improved her emotional outlook:

“It does make a difference when it comes to the symptoms … that you feel like if you can just get a handle on it, how you can just thrive.”

When discussing overall satisfaction with oral antipsychotic medications, a man with SZ stated:

“The symptoms are manageable. I still run into symptoms from here and there. But I’m really happy with the medication.”

Additional participant quotes pertaining to the effectiveness of, and satisfaction with, their antipsychotic medication are presented in Table 5 for participants with SZ and in Table 6 for participants with BD-I.

**Table 5:** Impacts of antipsychotic medications among participants with schizophrenia^a^

Favourable Impacts	Unfavourable Impacts
**Medication effectiveness**	**Side effects**
“I feel more focus and more level … and you also communicate better. I don’t get agitated.”	“I didn’t say anything until I ended up with diabetes. Because I was wondering why my hands were always so numb and my toes. So then I went for a physical and they was like, ‘Oh you have diabetes.’”
“I’m able to talk to people without feeling like something’s going to happen. Basically having a phone conversation, I’m able to do that, because I’d let the phone ring and let it go to voicemail and be done with it. But I’m able to talk on the phone, interact more.”	“It had me walking [in] slow motion, like I felt like I was a zombie or something.”
“Except for an occasional hallucination in a very high stress situation, I’m clear of symptoms as far as I can tell.”	“Because when you take it, it makes you eat. And then I guess it makes you feel like that’s a comfort. I guess because your mind isn’t racing and then constantly you need to do something and food is something you can do. I was cooking all kinds of things and eating whole course meals. I would eat everything.”
	“With every medicine I’ve been on, like, they’re affecting me sexually, too. I hate that side effect.”
	“You have a big appetite. A way big appetite.”
**Satisfaction with medication**	**Discontinuing or switching treatments**
“The medication I’m on now is right for me. So I feel like I’m going more and more into my recovery.”	“I think that I’m feeling better, that I don’t need it.”
“I used to go off [with anger] for no reason, but since I’ve been taking my medicine, I love being around people.”	“I take it now because, each time [I stopped], I’d end up in the hospital not too long after. And I don’t want to go there. I don’t feel like going back to the psychiatric ward.”

^a^Responses edited for clarity

**Table 6:** Impacts of antipsychotic medications among participants with bipolar I disorder^a^

Favourable Impacts	Unfavourable Impacts
**Medication effectiveness**	**Side effects**
“I feel like they help you to get control [of symptoms].”	“I’ve changed medications because of the weight gain.”
“Suicidal thoughts. I don’t have any suicidal thoughts at all.”	“Mine was low libido. And it’s causing issues in my marriage.”
	“Then you gain the weight and then you bring yourself back into a tailspin because you’re gaining weight
	“It [antipsychotic medication] drained my energy level completely.”
**Satisfaction with medication**	**Discontinuing or switching treatments**
“Right now, I’m good. Don’t switch [my medication]. Don’t switch me.”	“Diabetes runs in my family and I didn’t want to get [to] that point so I was like, ‘I can’t be on this medication.’”
“I haven’t been hospitalised since 2001. Been on the same medication since 2001.”	“Medication changes are horrible. I hate when the medication is working and you have to find something else. I have an appointment with my psychiatrist next week, and we’re going to have to find something else, because I can’t sleep 12 h every day.”

^a^Responses edited for clarity

### Unfavourable impacts of oral antipsychotic medications

A prominent theme among participants with SZ or BD-I was the unfavourable side effects of their oral antipsychotic medications, leading them to discontinue or switch treatments. Illustrative quotes on this theme from participants with SZ are presented in Table 5, whereas those for participants with BD-I are presented in Table 6.

#### Side effects

**Fig. 1:**
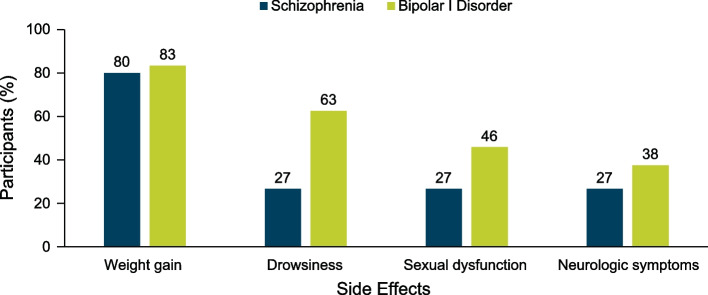
Most Common Side Effects of Antipsychotic Medications, as Reported by Focus Group Participants

Participants with SZ or BD-I described 36 or 34 discrete antipsychotic-associated side effects, respectively, that were burdensome to their daily life during the use of previous or current oral antipsychotics. Weight gain was the most reported side effect in participants with SZ (*n* = 12; 80%) and in those with BD-I (*n* = 20; 83%) (Fig. 1).

Among participants with SZ, 4 (27%) reported gaining between 50 and 100 pounds. Participants described health conditions related to their antipsychotic-associated weight gain, including diabetes, heart problems, hyperlipidaemia, hypertension, vision problems, physical numbness, sweating, and frequent urination. Antipsychotic-associated weight gain was described by a woman with SZ as detrimental to her health:

“I gained a lot of weight … it was just putting my health at risk.”

Reported weight gain in participants with BD-I ranged from 6 to 200 pounds, with 4 (17%) participants gaining between 40 and 75 pounds, and 3 (13%) gaining at least 75 pounds. Weight gain was attributed primarily to oral antipsychotic medications, and participants cited factors that contributed to their increased weight when taking antipsychotics, including inactivity, increased appetite, food cravings, impulsive spending, addictive behaviour, and low energy. A woman with BD-I described how her inactivity and appetite led to weight gain:

“It’s your low energy level and like increased appetite and sex, all of that just … yeah, there’s nothing. You’re just sitting there and you’re snacking and you’re sleeping.”

While weight gain was experienced with similar frequency across participants with SZ or BD-I, others were experienced at higher rates in participants with BD-I compared with those who had SZ (Fig. 1). These included drowsiness, sexual dysfunction, and neurologic symptoms, such as tremors, tics, or involuntary movements; these symptoms were described as problematic to daily functioning by many participants with BD-I, as described below:

“That’s one of the worst things is the sleepiness that you get taking some of these medications here and trying to drive an automobile in Atlanta traffic.”

When discussing sedation due to antipsychotic medications, a woman with BD-I stated:

“My medicine just knocks me out, and I’m tired all the time.”

Some participants expressed that they were physically and mentally uncomfortable with the sexual and neurologic side effects caused by their antipsychotic medication. A man with SZ stated:

“With every medicine I’ve been on, like they’re affecting me sexually, too. I hate that side effect.”

One woman with SZ discussed her experience with the neurologic side effects associated with taking her antipsychotic medication:

“I get [muscle tremors]. My hands will shake and I – especially like my diaphragm … will just go into spasms. So I don’t like that.”

#### Discontinuing oral antipsychotic medications

High rates of discontinuing or switching antipsychotic medications to avoid side effects were reported by focus group participants. Thirteen participants with SZ (87%) discontinued their antipsychotic medication at some point, either with or without consulting their MHCP. Additionally, 8 (53%) switched medications to avoid side effects. Six participants (40%) indicated that they would stop and/or had stopped taking a medication if it resulted in significant weight gain. A man with SZ discussed how weight gain influenced medication discontinuation:

“I had the same experience of extreme weight gain, tachycardia, which means I had to [stop taking it] …”

Sixteen participants (67%) with BD-I disclosed that they had discontinued their antipsychotic medication at some point, and 13 (54%) did so without consulting their MHCP. Overall, for participants with BD-I, antipsychotic medication side effects were a common reason for discontinuing treatment (*n* = 11; 46%). In addition, 11 participants (46%) specifically cited weight gain as a reason for discontinuing or switching medications. A woman with BD-I explained how her family history of diabetes led her to discontinue her antipsychotic medication:

“Diabetes runs in my family and I didn’t want to get [to] that point so I was like, ‘I can’t be on this medication.’”

## Discussion

This qualitative, cross-sectional study captured perspectives on disease burden and treatment experiences of people living with SZ or BD-I. Participants described negative impacts to relationships and emotional health due to symptoms of their disease. Disease-associated symptoms of SZ or BD-I had a substantial impact on the ability to work, and many participants noted having encounters with law enforcement during episodes of their respective illness. Participants also described the stigma of being labelled as mentally ill and how it negatively affected their self-esteem and their ability to maintain relationships.

Overall, participants with SZ or BD-I reported that oral antipsychotic medications were effective at managing their symptoms. Despite this, a prominent theme encountered in both the SZ and BD-I focus groups was that antipsychotic medications were associated with burdensome side effects. The most common side effects reported were weight gain, drowsiness, sexual dysfunction, and neurologic symptoms. Participants frequently remarked on the negative impact of side effects on daily function and quality of life, including the development of medical comorbidities, strained relationships, and issues with self-esteem. These findings align with recent surveys of patient perspectives on the impacts of disease burden and antipsychotic-associated side effects [21, 22].

Given that SZ and BD-I were associated with stigma and negative impacts on self-esteem, the physical and emotional toll of side effects may add to disease burden. Indeed, participants reported that the burdensome side effects of antipsychotic treatment often led to discontinuing or switching medications. Although health care providers were consulted before antipsychotic discontinuation in some cases, these decisions were made unilaterally by participants in other cases.

These results are consistent with previous studies of patient perspectives on the side effects associated with antipsychotic medications. Overall, antipsychotics are effective in managing SZ symptoms [32] and manic or mixed episodes of BD-I [33], but patient attitudes toward medications are mixed [34]. Understanding patient attitudes about antipsychotic medications is important because these attitudes influence treatment adherence and quality of life [35, 36]. Recent patient-focused research on the side effects associated with antipsychotic medications suggests that weight gain, drowsiness, and neurologic symptoms are burdensome and impact an individual’s social functioning, medication adherence, and work life [21, 22]. Suboptimal adherence to and discontinuation of antipsychotic medications increase the risk of negative clinical outcomes, including relapse and hospitalisation [16, 24, 25]. Previous research has found that side effects are a common reason for discontinuing treatment [21, 22]. Thus, our data highlight the potential benefits of antipsychotic medications that are effective at controlling the symptoms of SZ and BD-I while mitigating the burden of associated side effects. This research also underscores the trade-offs that patients often experience during antipsychotic treatment, where the degree of efficacy may influence the degree to which side effects are tolerated. Future research should explore these trade-offs in more detail. In addition, by using a semi-structured focus group approach, the current study generated a nuanced view of patient experiences with antipsychotic medications and the impact that treatment-associated side effects have on their physical health and daily functioning. Finally, this work is consistent with the emphasis that the US FDA has placed on reporting patients’ lived experience and the treatment outcomes that are most meaningful to them [37].

Focus groups have several methodological strengths. They allow participants to explore issues that are important to them and can be effective at eliciting subjective perspectives that may not be expressed in individual interviews or captured by conventional psychometric assessments [27]. In addition, focus groups help to facilitate discussion about sensitive topics while reminding participants of other symptoms or impacts [27]. Engaging authentically with participants in qualitative research can be challenging. It can be especially difficult when interviewing participants with a serious mental illness. The strategies and procedures implemented in this study aimed to minimise these challenges. For example, owing to the unpredictability of symptoms, participants were over-recruited to ensure an adequate sample size in case some participants did not attend on their scheduled day. Safety was prioritised by the presence of a clinical nurse to discuss any concerns participants might have during the session. Additionally, moderators trained in qualitative research used conversational interview techniques to build rapport and show empathy, and the moderator guide was structured to cover the most important concepts first. During the focus groups, care was taken by the moderators to paraphrase responses so that participants’ perspectives of their symptoms and experiences were captured accurately.

### Limitations

The small sample size (15 participants with SZ and 24 with BD-I) inherent to focus groups may limit the generalisability of these results to the larger populations of people living with SZ or BD-I. Because the diagnoses of SZ or BD-I were based on self-report, they were not confirmed. In addition, individuals with serious mental illness who participate in research studies, such as focus groups, may have different attitudes towards their illness and treatment compared with the larger population of individuals with serious mental illness who do not participate in research studies. Also, focus group discussions may generate collaborative responses. Therefore, not every participant answered every question posed during the focus groups. Thus, analysis relied on the coding of both verbal and nonverbal responses. Another limitation is that it may be difficult for participants to separate the side effects of oral antipsychotic medications from those of other medications used to treat their illness, including mood stabilisers, antidepressants, and/or anxiolytics.

## Conclusions

Participants in this study discussed a range of experiences with oral antipsychotic medications taken for the treatment of SZ or BD-I, providing a better understanding of patient treatment experiences. Although favourable impacts of medication on disease-related symptoms were reported, participants in both groups highlighted burdensome side effects of treatment that negatively impacted their quality of life. Patients’ perspectives on these impacts and side effects of oral antipsychotic medications underscore the need to reduce treatment burden for individuals living with SZ or BD-I and highlight a need for new medications that are effective and reduce clinical burden while improving treatment adherence and patient outcomes. Study results describe the burden that disease symptoms and antipsychotic medication side effects have on patients, including their impact on quality of life. Learning from and understanding these patient experiences may lead to treatment choices that improve the benefit-to-risk ratio for the individual with severe mental illness.

## Data Availability

The data collected in this study are proprietary to Alkermes, Inc. Alkermes, Inc., is committed to public sharing of data in accordance with applicable regulations and laws, and requests can be submitted to the corresponding author.

## Abbreviations

BD-I: Bipolar I disorder
MHCP: Mental health care professional
SD: Standard deviation
SZ: Schizophrenia
